# Local electrogram delay recorded from left ventricular lead at implant predicts response to cardiac resynchronization therapy: Retrospective study with 1 year follow up

**DOI:** 10.1186/1471-2261-12-34

**Published:** 2012-05-20

**Authors:** Rostislav Polasek, Pavel Kucera, Pavel Nedbal, Tomas Roubicek, Tomas Belza, Jana Hanuliakova, David Horak, Dan Wichterle, Josef Kautzner

**Affiliations:** 1 Department of Cardiology, Regional Hospital Liberec, Husova 10, Liberec, Czech Republic; 2 Department of Cardiology, Institute for Clinical and Experimental Medicine, Videnska 1958/9, Prague, Czech Republic

**Keywords:** Cardiac resynchronization therapy, Reverse remodelling, LV lead location, Electrical dyssynchrony

## Abstract

**Background:**

Considerable proportion of patients does not respond to the cardiac resynchronization therapy (CRT). This study investigated clinical relevance of left ventricular electrode local electrogram delay from the beginning of QRS (QLV). We hypothesized that longer QLV indicating more optimal lead placement in the late activated regions is associated with the higher probability of positive CRT response.

**Methods:**

We conducted a retrospective, single–centre analysis of 161 consecutive patients with heart failure and LBBB or nonspecific intraventricular conduction delay (IVCD) treated with CRT. We routinely intend to implant the LV lead in a region with long QLV. Clinical response to CRT, left ventricular (LV) reverse remodelling (i.e. decrease in LV end-systolic diameter - LVESD ≥10%) and reduction in plasma level of NT-proBNP >30% at 12-month post-implant were the study endpoints. We analyzed association between pre-implant variables and the study endpoints.

**Results:**

Clinical CRT response rate reached 58%, 84% and 92% in the lowest (≤105 ms), middle (106-130 ms) and the highest (>130 ms) QLV tertile (p < 0.0001), respectively. Longer QRS duration (p = 0.002), smaller LVESD and a non-ischemic cardiomyopathy (both p = 0.02) were also univariately associated with positive clinical CRT response. In a multivariate analysis, QLV remained the strongest predictor of clinical CRT response (p < 0.00001), followed by LVESD (p = 0.01) and etiology of LV dysfunction (p = 0.04). Comparable predictive power of QLV for LV reverse remodelling and NT-proBNP response rates was observed.

**Conclusion:**

LV lead position assessed by duration of the QLV interval was found the strongest independent predictor of beneficial clinical response to CRT.

## Background

Cardiac resynchronization therapy (CRT) has become an established treatment strategy in patients with advanced chronic systolic heart failure and a wide QRS complex. There is an overwhelming evidence suggesting that CRT improves exercise tolerance and the quality of life, prevents hospitalizations for heart failure and has a favorable impact on prognosis [1-5]. Unfortunately, approximately 30% of patients fail to respond clinically to CRT [6]. This figure is even higher when LV reverse remodelling is chosen as an endpoint for the response [6].

The possible reasons for non-responsiveness to CRT include the amount of nonviable myocardium [7,8], natural progression of the underlying disease and a type of the left ventricular (LV) conduction abnormality – i.e. right bundle branch block (RBBB) [9-11]. In addition, response to CRT seems to depend on the LV lead positioning and apical or anterolateral pacing sites were found predictive of a poor outcome [12].

As CRT aims principally to correct electrical ventricular dyssynchrony, optimal position of the LV lead could be expected in the LV region with the most delayed spontaneous depolarization. Based on our previous mapping studies in patients with LBBB [13], such regions are predominantly located at the lateral wall, close to the base of the heart. To insert the LV lead as close as possible to the latest activated region, we measured the QLV defined as an interval between the beginning of the QRS complex and the LV electrode local electrogram. During implantation, the QLV interval was maximized whenever possible. We hypothesized that the longer QLV will be associated with the higher probability of resynchronization and positive CRT response.

## Methods

### Patient population

We retrospectively analyzed data of all patients with implanted biventricular pacemaker or defibrillator at the Liberec Regional Hospital, Czech Republic between June 2005 and June 2010. CRT was indicated according to the guidelines of the European Society of Cardiology [14]. In this period, standardized echocardiographic examination and biochemical assessment of heart failure were routine parts of preimplant and one-year postimplant check-ups at our institution. Only patients with LBBB or IVCD were evaluated.

A total of 214 non-RBBB patients were implanted with the CRT system within the above-mentioned period. After analysis of all available data, 53 subjects were excluded from the study. The exclusion criteria were as follows: 1) death from other cause than heart failure during the first 12 months of the follow-up (n = 6), 2) insufficient electrophysiological data from the implant procedure (n = 35), 3) significant change in post-implantation clinical status (e.g. coronary revascularization or acute myocardial infarction) (n = 7), 4) unavailability of a patient for follow-up (n = 2), 5) ventricular non capture (n = 3).

All patients signed an informed consent with the procedure. The study was performed in accordance with the guidelines proposed in the Declaration of Helsinki and the analysis was approved by Ethics Committee of the Regional Hospital in Liberec, reference number EK/158/2011.

### Implant procedure

Commercially available CRT systems were implanted by two operators. The right ventricular lead was commonly placed in the midseptum region. The LV lead was inserted transvenously with a preference for lateral cardiac veins, followed by posterolateral or anterolateral position. Whenever possible, attempts were made to maximize QLV interval at implant, i.e. when the LV lead electrogram was not recorded in the terminal part of the QRS, other available veins were explored in order to achieve the longer QLV. In cases when the QLV interval was significantly longer in a proximal part of a vein, all efforts were made to provide stability of the lead in this position (sometimes changing the lead design).

Empiric atrioventricular delay of 120 ms and V-V delay 0 ms were programmed at implant and were not routinely optimized. When no clinical improvement was observed during early follow- up visit, at least one echocardiographic-guided optimization of these intervals was performed.

### Electrophysiological measurements

**Figure 1:**
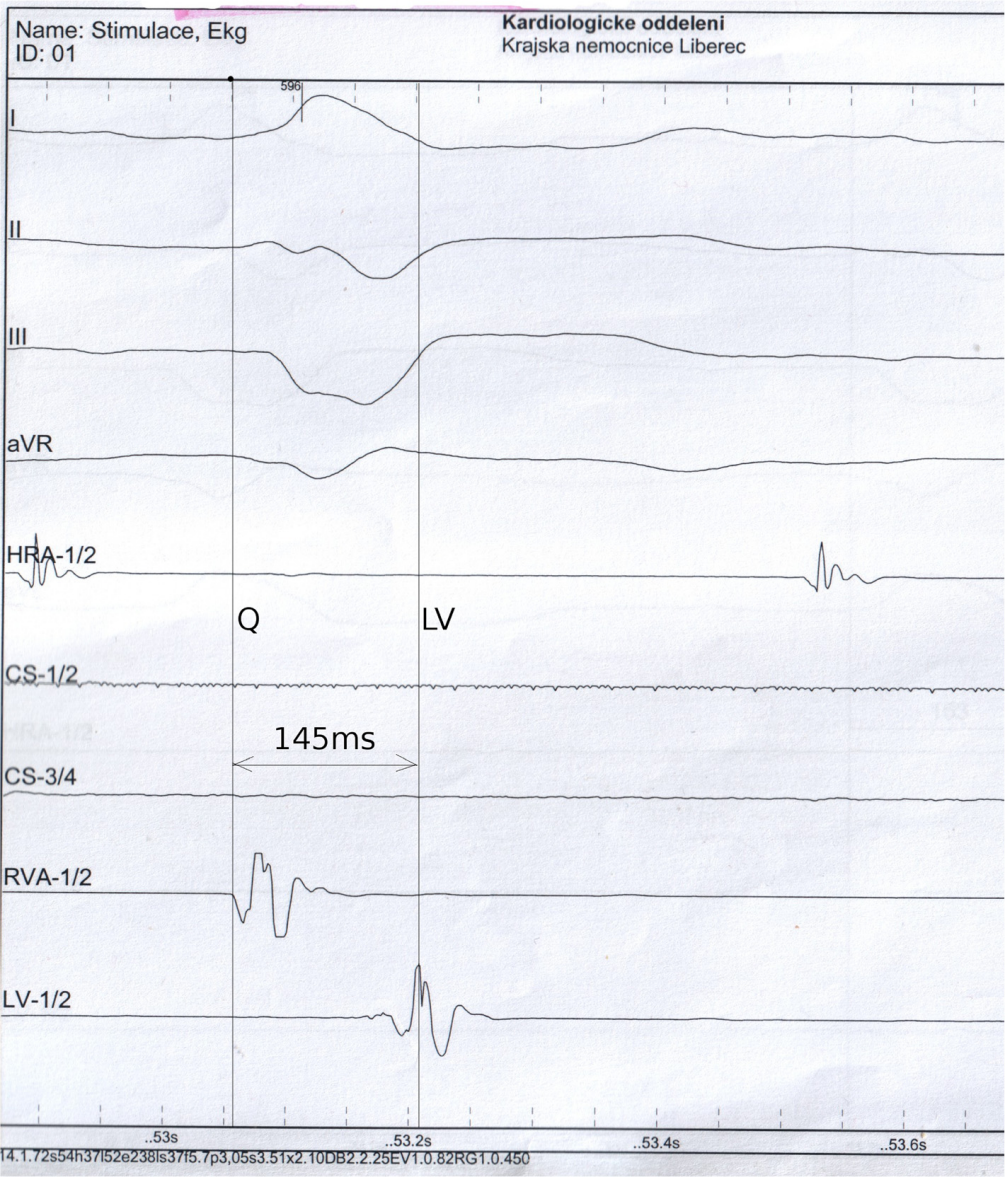
**Measurement of the QLV.** Printout of the electrophysiological recording system at 200 mm/s paper speed showing the interval from the beginning of the native QRS complex to the local electrogram from the LV lead. Labels: Lead II, III, aVR of the surface ECG; RVA-1/2 - right ventricular electrogram; LV-1/2 - LV electrogram; HRA 1/2 - right atrial electrogram.

The QLV interval was measured from the beginning of the native QRS complex to the first sharp spike of LV electrogram (Figure 1). After the final position of the left ventricular lead was reached, the local bipolar electrogram from the tip of the LV lead was displayed simultaneously with a surface ECG at sweep speed of 200 mm/s on the electrophysiological recording system (Biotronik EP Control, Germany). QLV was measured by technician using an electronic caliper. The recordings were carefully visually inspected and manually edited, when necessary, by a single observer (dedicated physician). Print outs of all patients were archived. The QLV ratio, defined as QLV divided by QRS duration (QRSd), was also assessed.

### Echocardiographic examination

LV end systolic diameter (LVESD), LV end diastolic diameter (LVEDD) measured in M-mode and LV ejection fraction (LVEF) assessed by planimetry were collected at baseline and 12-month later using scanners of one manufacturer (Philips, Amsterdam, The Netherlands). Measurements were performed according to the recommendations of American Society of Echocardiography [15] by one operator who was blinded to the QLV values. A relative LVESD reduction of ≥10% was considered clinically significant.

### Biomarker assessment

Plasma levels of N-terminal pro-hormone of brain natriuretic peptide (NT-proBNP) were assessed before implant and at 12-month follow-up using commercially available assay (Roche, Switzerland). A clinically meaningful decrease of >30% was considered significant. In 24 patients NT-proBNP datasets were not available.

### Study objectives

The primary endpoint of the study comprised positive clinical response to CRT at 12 months defined as an improvement of the functional status by at least 1 NYHA class and/or LV reverse remodelling (LVESD reduction of ≥10%). Patients who died of heart failure within one year after implantation were considered non-responders. Signs of LV reverse remodelling alone and NT-proBNP response were the other endpoints of the study.

### Statistical analysis

Continuous variables were expressed after testing for normality of distribution (Shapiro Wilk’s test) as the means with standard deviations and compared with the 2-tailed *t*-test for dependent or independent samples, as appropriate. Non-normally distributed variables were expressed as medians and interquartile range and compared by Mann–Whitney *U* test or Wilcoxon’s paired test, as appropriate. Categorical variables were expressed as percentages and compared by *χ*^2^-test. Relationship between variables (or their change) was assessed by Pearson’s correlation analysis. ANOVA with Scheffe’s post hoc test was used for the analysis of CRT response rate in subgroups defined by tertiles of individual baseline variables. Multivariate regression analysis that included all univariately significant factors was used to test the association of CRT response rate with baseline clinical, echocardiographic, and electrophysiological variables. A p-value <0.05 was considered significant. All analyses were performed using the STATISTICA vers. 6.1 software (Statsoft, Inc.).

## Results

The baseline characteristics of the study population are shown in Table 1. During 12 months of follow-up, 3 patients died due to progressive heart failure and were assigned to the non-responder subgroup. A total of 124 (77.0%) and 37 (23.0%) patients were classified as clinical responders and non-responders, respectively. There were 94 (58.4%) LV remodelling responders and 89/137 (65.0%) NT-proBNP responders.

**Table 1:** Baseline characteristics (n = 161)

	
Age (years)	67.0 ± 9.4 (32 – 86)
Female (%)	20.5
Ischemic cardiomyopathy (%)	53.4
NYHA functional class	3.1 ± 0.5 (2 – 4)
- NYHA II (%)	8.1
- NYHA III (%)	70.8
- NYHA IV (%)	21.1
LVEF (%)	24.7 ± 5.1 (15 – 35)
LVESD (mm)	57.1 ± 8.2 (37 – 90)
LVEDD (mm)	66.4 ± 7.6 (45 – 96)
Mitral regurgitation (grade)	1.9 ± 1.1 (1 – 4)
NT-proBNP (pg/ml)	4221 ± 5563 (270 – 35000)
Atrial fibrillation (%)	14.3
ICD (%)	67.1
QRSd (ms)	157 ± 20 (120 – 211)
QLV (ms)	117 ± 28 (65 – 189)
QLV ratio	0.74 ± 0.12 (0.46 – 0.95)
Baseline medication	
- Beta-blockers (%)	96
- ACEI or ARB (%)	99
- Loop diuretics (%)	91
- Aldosterone antagonists (%)	89

**Figure 2:**
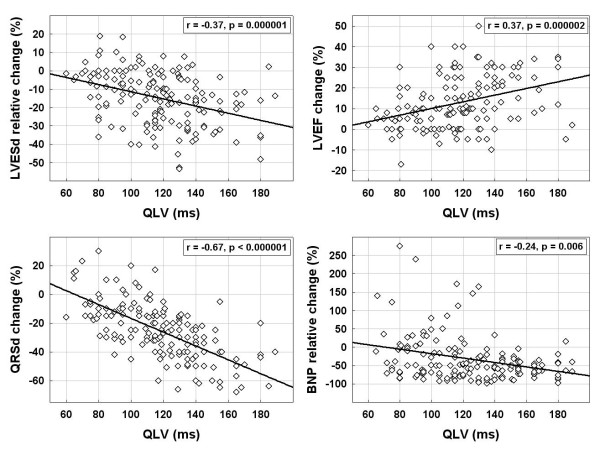
**Relationship of the QLV and CRT effects.** The greater QLV at implantation of CRT system correlates with an increase in LVEF, decrease in LVESD, shortening of QRSd, and reduction in NT-proBNP at 12-month follow-up. Pearsons’s correlation coefficients (r) with p-values are provided. Abbreviations as in Table 1.

Only exceptionally (n = 4), responders were identified by LV remodelling alone, i.e. without improvement in NYHA class. Baseline differences between responders and non-responders are shown in Table 2. Responders presented more frequently with non-ischemic cardiomyopathy, had less dilated left ventricle and wider QRS complex. The greatest difference at implant was observed both for the QLV interval and QLV ratio (123 ± 26 ms vs. 98 ± 27 ms, and 0.76 ± 0.11 vs. 0.66 ± 0.14, respectively, both p < 0.00001). At 12-month follow-up, responders differed significantly from non-responders in NYHA class, LVEF, LV diameters, QRS duration and NT-proBNP level. The QLV correlated weakly but significantly with an increase in LVEF, decrease in LVESD, shortening of QRSd and reduction of NT-proBNP induced by CRT (Figure 2).

**Table 2:** Baseline difference between responders and non-responders

	**Responders (n = 124)**	**Non-responders (n = 37)**	**P value**
Age (years)	66.7 ± 9.1	67.9 ± 10.5	0.51
Female (%)	21.8	16.2	0.46
Ischemic cardiomyopathy (%)	48.4	70.3	0.02
Atrial fibrillation (%)	13.6	16.2	0.70
NYHA class	3.1 ± 0.5 median 3 (IQR 3–3)	3.2 ± 0.6 median 3 (IQR 3–4)	0.52
Baseline LVEF (%)	24.9 ± 5.1 median 25 (IQR 20–30)	23.8 ± 5.3 median 23 (IQR 20–30)	0.27
LVESD (mm)	56.3 ± 8.2	60.1 ± 7.6	0.02
LVEDD (mm)	65.7 ± 7.6	68.7 ± 7.0	0.04
Mitral regurgitation grade	1.9 ± 1.1 median 2 (IQR 1–3)	1.9 ± 1.1 median 2 (IQR 1–3)	0.95
NT-proBNP (pg/ml)	4284 ± 5862 median 2492 (IQR 1535–4800)	4002 ± 4444 median 2700 (IQR 1898–4758)	0.57
QRSd (ms)	160 ± 20	147 ± 19	0.0006
QLV (ms)	122.8 ± 25.7	98.2 ± 27.0	0.000002
QLV ratio	0.76 ± 0.11	0.66 ± 0.14	0.000007

The values are mean ± standard deviation, median (IQR, interquartile range) or proportions. Abbreviations as in Table 1.

**Figure 3:**
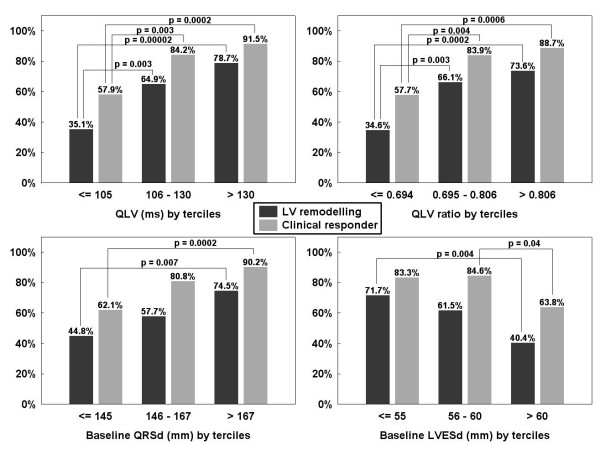
**CRT responder rates in subgroups defined by tertiles of baseline variables.** Response rates in percentages when population was categorized by tertiles of the QLV, QLV ratio, QRSd, and LVESD. Grey bars indicate clinical response to CRT and black bars proportion of patients who showed reverse LV remodelling. Abbreviations as in Table 1.

Among the baseline categorical variables, only ischemic etiology of cardiomyopathy was significantly associated with lower clinical CRT response rate (69.8 vs. 85.3%, p = 0.02) and with less reverse LV remodelling (48.8 vs. 69.3%, p = 0.008) as compared with non-ischemic cardiomyopathy. When continuous baseline variables were categorized by tertiles (middle tertile cut-off points for the QLV, QLV ratio, QRSd and LVESD were 105 - 130 ms, 0.694 - 0.806, 145 - 167 ms, and 55 - 60 mm, respectively), significant association with clinical CRT response was found for the baseline QLV (p = 0.00005), QLV ratio (p = 0.0002), baseline QRSd (p = 0.002), and LVESD (p = 0.02). Similarly, significant association with reverse LV remodelling was found for the baseline QLV (p = 0.00001) and QLV ratio (p = 0.00007), baseline QRSd (p = 0.007), and LVESD (p = 0.004). Response rates in individual subgroups and their comparison are shown in Figure 3. NT-proBNP response rates were 49% vs. 85% (p = 0.002) in lower vs. upper tertile of QLV, respectively. Other baseline factors (age, gender, basic heart rhythm, LVEF, NYHA class, grade of mitral regurgitation and NT-proBNP level) were not significantly (p > 0.20) associated with clinical CRT response and were not subjected to multivariate analysis.

Table 3 shows detailed results of univariate and multivariate association between baseline factors (analyzed as continuous variables) and clinical CRT response, LV remodelling and NT-proBNP response. Because of interdependence, the QLV and QLV ratio were entered separately into the two linear regression models. Similarly, because of the strong interaction between LVESD and LVEDD, only LVESD, which was more tightly associated with study endpoints, entered into both models. When a stepwise forward analysis was applied, the QLV and QLV ratio were comparably the most powerful and independent predictors of study endpoints. LVESD and etiology of cardiomyopathy appeared to be weaker but still significant covariates. NT-proBNP response was generally less predictable by baseline characteristics but again the QLV and QLV ratio remained the strongest independent factors. When multivariate analysis was performed separately in ischemic and non ischemic subpopulations, the association between the QLV and CRT effects was preserved in both subgroups.

**Table 3:** Univariate and multivariate association between baseline factors and clinical, echocardiographic and biochemical CRT response

	**Univariate analysis**			**Multivariate analysis (MODEL 1)**			**Multivariate analysis (MODEL 2)**		
**Baseline variable**	**Clinical response**	**LV remodelling**	**BNP response**	**Clinical response**	**LV remodelling**	**BNP response**	**Clinical response**	**LV remodelling**	**BNP response**
ICM/NICM	0.02	0.008	0.04	0.04	0.01	Ns	0.046	0.02	ns
QLV	<0.00001	<0.00001	0.0001	<0.00001	<0.00001	0.0001	-	-	-
QLV ratio	<0.00001	<0.00001	0.001	-	-	-	0.0004	<0.00001	0.01
QRSd	0.0005	0.002	0.003	ns	ns	Ns	0.04	ns	0.04
LVESD	0.01	0.0006	0.12	0.009	0.0002	Ns	0.01	0.0003	ns
LVEDD	0.03	0.0007	0.19	-	-	-	-	-	-

The figures are p-values for linear regression between baseline variables and CRT response rates analyzed in two multivariate models that included either QLV (Model 1) or QLV ratio (Model 2). ICM – ischemic cardiomyopathy; NICM – non-ischemic cardiomyopathy. Other abbreviations as in Table 1.

A mutual combination of the QLV, LVESD, and QRSd was used for further stratification of CRT effects. Patients who belonged either to the “high-risk” tertiles of QLV ≤ 105 ms and/or LVESD > 60 mm presented with lower CRT response rate of 64% (n = 80). Among them, 47 patients had ischemic cardiomyopathy and had even lower response rate of 57%. In a complementary subpopulation, i.e. in those with both QLV > 105 ms and LVESD ≤ 60 mm, the clinical CRT response reached 92% (n = 81). Among them, 41 patients had non-ischemic cardiomyopathy and had even greater CRT response of 98%. On the other hand, CRT response rate was only 52% in patients with both QLV ≤ 105 ms and LVESD > 60 mm (n = 23). Among them, 14 patients had ischemic cardiomyopathy and even lower response rate (36%).

## Discussion

This study represents another piece of evidence on association between response to CRT and the LV lead position, expressed as the QLV interval. Patients in the lowest QLV tertile (QLV ≤ 105 ms) had significantly lower probability to become clinical responders or to show LV reverse remodelling at 12-month follow-up. On the contrary, the best clinical response to CRT (92% of subjects) was observed in patients with both QLV > 105 ms and LVESD ≤ 60 mm. Clinical relevance of the QLV interval was further confirmed by its correlation with the change in plasma levels of biochemical marker of heart failure. All these findings support the hypothesis that pacing within the late activated LV region has the highest potential to correct electrical dyssynchrony and associated LV mechanical dysfunction in patients with intraventricular conduction abnormality of the LBBB type.

### Comparison with other studies

Similar to our study, Singh et al [16]. analyzed clinical response after one year of CRT in patients with LBBB pattern. The authors found QLV/QRSD < 0.5 in significant proportion of cases (27 of 71) and these subjects presented with increased mortality and hospitalization rate for heart failure. The proportion of such cases was significantly lower (5 cases only, i.e. 3.1%) in our study. This difference may reflect the fact that we attempted to maximize QLV at implant and used wider spectrum of LV lead designs to achieve this goal.

Our results are also in concordance with two other recently published studies. The retrospective analysis by Fatemi et al [17]. reported similar observation in a smaller group of CRT patients (n = 72) with longer follow-up (30 months). The authors found correlation of the QLV with LV reverse remodelling and/or clinical response. The other study [18] was derived from the multicenter SMART AV Trial comparing different methods of atrioventricular delay optimization strategy among CRT subjects. In a prospectively designed substudy, the response to CRT was assessed in 426 patients with a 50% rate of reverse remodelling (change in LV end systolic volume) after 6 months of follow-up. Similar to our results, higher proportion of reverse remodelling was found in subjects with longer QLV. Based on their results, the authors recommended for positioning the LV lead to reach the QLV at least 95 ms. However, there are some important differences between our trial and the two aforementioned studies that have to be emphasized. First, the patient populations differed substantially. In both other studies, patients with RBBB were enrolled. In the study by Fatemi et al., patients with scar in the lateral wall were excluded. In SMART AV, there was a higher proportion of women (34 vs. 20% in our study) and fewer patients were in NYHA IV (3 vs. 20%). The follow-up in SMART AV Trial was significantly shorter (6 months versus 12 months). Second, none of the other studies attempted to maximize the QLV already at implant.

In contrast to the two above mentioned trials [17,18] and contrary to general expectation, we observed higher proportion of clinical responders and LV reverse remodelling in our CRT population. One plausible explanation could be the exclusion of RBBB patients who are known to have less benefit from CRT [9-11] from our study. Another reason, at least in comparison with the SMART AV Trial, could be the longer follow-up in our study that might allow reverse remodelling in more patients. The third possible explanation, which seems to be the most important, is our strategy to place the LV lead at the site with the latest possible activation. This practice reflects our earlier experience with electroanatomic mapping of late activated regions and resulted in longer median QLV interval in our cohort (116 ms) and narrower interquartile range (IQR) of 40 ms (95-135 ms) in contrast to the SMART AV study with median QLV of 95 ms and IQR of 50 ms (70-120 ms). Preserved predictive power of the QLV even after a priori optimization resulting in narrower range of QLV further underlines its clinical utility. It also has to be emphasized that the method of QLV measurement differed in both studies. In SMART AV substudy [18], measurements were performed from the device programmers using a sweep speed of 100 mm/s. This strategy required correction for the average variable latency (or noise) between the alignment of surface ECG and the electrogram channels in these devices. In contrast, our measurements were performed using electrophysiological recording system with a simultaneous display of LV electrograms and surface ECG channels at 200 mm/s sweep speed.

### Study implications

We acknowledge that to dichotomize the effect of CRT responder vs. non-responder is an oversimplification. The response to CRT is a continuum with patients who worsen on one side and hyper-responders on the other. However, significant correlation between the QLV and LV reverse remodelling suggests that achieving higher QLV value at implant means not only higher likelihood of a positive clinical and echocardiographic response to CRT but also the greater magnitude of the effect. Thus, our results clearly imply that the QLV should be maximized during the LV lead implant as much as possible. On the contrary, patients with QLV ≤ 105 ms are less likely to show positive response to CRT.

We also assessed predictive power of another parameter, QLV ratio that related the QLV to QRSd. The prediction characteristics of QLV ratio for response to CRT appeared somewhat inferior to the QLV interval. Obviously, this is because the QLV interval conveys not only information on the optimal lead position but also reflects the QRSd that is univarite predictor of CRT response per se. Such composite predictor has to be inherently better than QLV ratio that describes LV lead positioning only. Concordantly, QRSd became non-significant predictor of CRT effects when entered the multivariate model together with the QLV interval. For practical reasons, guiding the LV lead placement by readily available QLV interval can be recommended. On the other hand, QLV ratio (together with other clinical variables) rather than QLV interval might be preferred for optimization of CRT effects in future interventional CRT trials investigating alternative methods of LV pacing.

### Study limitations

This study has several limitations. First, it was a retrospective, single-center study and this may limit generalization of its results. Second, LV lead positions were not recorded routinely on cineloops and therefore, we could not evaluate correlation between the QLV and actual pacing site location. Third, the amount and the location of LV scar tissue that may interfere with the CRT response was not assessed and included in multivariate analysis. Fourth, we assessed LV reverse remodelling using one-dimensional parameter, specifically LVESD, because LV end-systolic volume (LVESV) was not available in all patients. This might influence to some extent the results on reverse remodelling as LVESV is more frequently used for this purpose. On the other hand, our dichotomy of ≥10% for LVESD corresponds to ≥21-22% for LVESV when estimated by Teichholz formula, i.e. our definition of LV reverse remodelling was clearly more stringent than that used, for example, in SMART AV substudy (≥15% for LVESV). The measurement of LV end-systolic volume is also less reproducible as compared with LVESD. Some studies have shown that inter- (<20%) and intra-individual (5-10%) variability of LV end-systolic volume is higher as compared with variability of LVESD (1-3% intra-individually) [12]. Finally, because we did not find relevant dichotomy for clinically significant NT-proBNP decrease in literature we arbitrarily used the cut-off point of >30% and made sure that alternative cut-off values provided comparable results.

## Conclusions

LV lead position assessed by the QLV interval was found the strongest independent predictor of beneficial response to CRT, followed by LVESD and etiology of LV dysfunction. In patients with long QLV (>130 ms), clinical CRT response and LV remodelling rates at one year after implant were higher by 58% and 124%, respectively, when compared with short QLV ≤ 105 ms. This association between the QLV and CRT effects was preserved both in ischemic and non-ischemic cardiomyopathy subgroups. Therefore, attempts to maximize the QLV during LV lead placement should be considered in all non-RBBB CRT patients. Clinical utility of this approach should be tested in prospective interventional trial.

## Competing interest

RP: none declared, PK: none declared, PN: none declared, TR: none declared, TB: none declared, JH: none declared, DH: none declared, DW: none declared, JK: received consulting fees or honoraria from Biotronik, Boston Scientific, Medtronic and St. Jude Medical. He is a recipient of research grants from Biotronik, Boston Scientific and Medtronic.

## Authors’ contributions

RP: conception and design of the research, acquisition of data, analysis and interpretation of the data, drafting of the manuscript, PK: acquisition of data, PN: acquisition of data, TR: acquisition of data, drafting of the manuscript, TB: acquisition of data, JH: drafting of the manuscript, DH: acquisition of data, DW: statistical analysis, critical revision of the manuscript for important intellectual content, JK: conception and design of the research, critical revision of the manuscript for important intellectual content, supervision. All authors read and approved the final manuscript.

## Pre-publication history

The pre-publication history for this paper can be accessed here:

http://www.biomedcentral.com/1471-2261/12/34/prepub
